# Comparative Studies in the A30P and A53T α-Synuclein *C. elegans* Strains to Investigate the Molecular Origins of Parkinson's Disease

**DOI:** 10.3389/fcell.2021.552549

**Published:** 2021-03-22

**Authors:** Michele Perni, Annemieke van der Goot, Ryan Limbocker, Tjakko J. van Ham, Francesco A. Aprile, Catherine K. Xu, Patrick Flagmeier, Karen Thijssen, Pietro Sormanni, Giuliana Fusco, Serene W. Chen, Pavan K. Challa, Julius B. Kirkegaard, Romain F. Laine, Kai Yu Ma, Martin B. D. Müller, Tessa Sinnige, Janet R. Kumita, Samuel I. A. Cohen, Renée Seinstra, Gabriele S. Kaminski Schierle, Clemens F. Kaminski, Denise Barbut, Alfonso De Simone, Tuomas P. J. Knowles, Michael Zasloff, Ellen A. A. Nollen, Michele Vendruscolo, Christopher M. Dobson

**Affiliations:** ^1^Department of Chemistry, Centre for Misfolding Diseases, University of Cambridge, Cambridge, United Kingdom; ^2^University Medical Centre Groningen, European Research Institute for the Biology of Aging, University of Groningen, Groningen, Netherlands; ^3^Department of Chemistry and Life Science, United States Military Academy, West Point, NY, United States; ^4^Department of Clinical Genetics, Erasmus University Medical Center, Rotterdam, Netherlands; ^5^Department of Applied Mathematics and Theoretical Physics, University of Cambridge, Cambridge, United Kingdom; ^6^MRC Laboratory for Molecular Cell Biology (LMCB) University College London, London, United Kingdom; ^7^Department of Chemical Engineering and Biotechnology, University of Cambridge, Cambridge, United Kingdom; ^8^MedStar-Georgetown Transplant Institute, Georgetown University School of Medicine, Washington, DC, United States; ^9^Department of Life Sciences, Imperial College London, London, United Kingdom

**Keywords:** *C. elegans*, Parkinson's disease, alpha-synuclein, drug discovery, protein aggregation, protein misfolding, neurodegenerative diseases, transgenic model

## Abstract

The aggregation of α-synuclein is a hallmark of Parkinson's disease (PD) and a variety of related neurological disorders. A number of mutations in this protein, including A30P and A53T, are associated with familial forms of the disease. Patients carrying the A30P mutation typically exhibit a similar age of onset and symptoms as sporadic PD, while those carrying the A53T mutation generally have an earlier age of onset and an accelerated progression. We report two *C. elegans* models of PD (PD_A30P_ and PD_A53T_), which express these mutational variants in the muscle cells, and probed their behavior relative to animals expressing the wild-type protein (PD_WT_). PD_A30P_ worms showed a reduced speed of movement and an increased paralysis rate, control worms, but no change in the frequency of body bends. By contrast, in PD_A53T_ worms both speed and frequency of body bends were significantly decreased, and paralysis rate was increased. α-Synuclein was also observed to be less well localized into aggregates in PD_A30P_ worms compared to PD_A53T_ and PD_WT_ worms, and amyloid-like features were evident later in the life of the animals, despite comparable levels of expression of α-synuclein. Furthermore, squalamine, a natural product currently in clinical trials for treating symptomatic aspects of PD, was found to reduce significantly the aggregation of α-synuclein and its associated toxicity in PD_A53T_ and PD_WT_ worms, but had less marked effects in PD_A30P_. In addition, using an antibody that targets the N-terminal region of α-synuclein, we observed a suppression of toxicity in PD_A30P_, PD_A53T_ and PD_WT_ worms. These results illustrate the use of these two *C. elegans* models in fundamental and applied PD research.

## Introduction

α-Synuclein (α-syn) is an intrinsically disordered protein expressed at high levels in the human brain, which in Parkinson's disease (PD) and related disorders aggregates to form Lewy bodies (Gómez Tortosa et al., 1998; Spillantini et al., 1998; Dawson and Dawson, 2003; Chiti and Dobson, 2006, 2017; Knowles et al., 2014; Dettmer et al., 2016). Because the aberrant assembly of α-syn is a common feature in the development of these diseases (Chiti and Dobson, 2006), intense efforts have been devoted toward understanding and inhibiting this phenomenon (Lee and Trojanowski, 2006; Tóth et al., 2014). Growing evidence shows that the formation of α-syn aggregates may be induced by aberrant protein-protein or protein-membrane interactions (Auluck et al., 2010; Galvagnion et al., 2015; Dettmer et al., 2016), by malfunctions of molecular chaperones (Witt, 2013; Cox et al., 2014), and by the effects of post-translational modifications (Fujiwara et al., 2002; Hasegawa et al., 2002; Saito et al., 2003; Bendor et al., 2013) and familial mutations in the α-syn gene (Li et al., 2001; Zarranz et al., 2004; Emmer et al., 2011; Sacino et al., 2013). The pathological phenotype of non-heritable idiopathic PD has been shown to be close to that associated with familial PD. However, familial forms of PD, which account for a 10–15% of all PD cases, can have a different age of onset, severity of the disease, and resistance to treatments (Kasten and Klein, 2013).

Among the disease-associated mutations (Li et al., 2001; Zarranz et al., 2004; Emmer et al., 2011; Sacino et al., 2013), the amino acid substitutions A30P and A53T (Li et al., 2001) have been shown to be linked with familial PD (Thomas and Beal, 2007). It has been observed that patients carrying the A30P mutation typically exhibit a similar age of onset and symptoms as sporadic PD, while those carrying the A53T mutation have an earlier age of onset and an accelerated progression of the disease (Polymeropoulos et al., 1997; Krüger et al., 2001; Schiesling et al., 2008). Biophysical studies have shown that these mutations significantly affect the *in vitro* mechanism of aggregation of α-syn (Flagmeier et al., 2016), and in particular, A53T α-syn was shown to aggregate more rapidly than the A30P or wild-type α-syn (Narhi et al., 1999; Li et al., 2001, 2002). Less agreement, however, exists as to whether the A30P variant aggregates more rapidly (Narhi et al., 1999; Li et al., 2001), at a similar rate (Lemkau et al., 2012) or more slowly (Conway et al., 2000), than the wild-type protein. Recently, we utilized a three-pronged strategy to characterize the influence of these mutations on the mechanism of the aggregation of α-syn *in vitro* (Flagmeier et al., 2016) and found that the rates of fibril amplification, but not of lipid-induced nucleation, were slightly enhanced in the case of the A30P variant, and were markedly increased in the case of the A53T mutant compared with the wild-type protein (Flagmeier et al., 2016). The importance of studying these mutational variants in animal models has been investigated using a variety of different animal models such as mice, fish or flies (Dehay et al., 2015; Jagmag et al., 2016; Visanji et al., 2016). In several transgenic mice lines, overexpressing human wild-type, A53T, or A30P α-synuclein showed high correlation with transgene expression, in combination with toxic gain of function mechanism for α-synuclein pathogenesis (Visanji et al., 2016). Overexpression of these genes can indeed lead to neurodegeneration, loss of striatal dopamine, and locomotors dysfunction (Dehay et al., 2015). Nevertheless, invertebrates such as *Drosophila* have also proven powerful very tools to investigate the molecular mechanisms of toxicity associated with α-syn aggregation (Mizuno et al., 2010) due to their 75% homology with human disease genes, rapid generation cycle (10–14 days), short life span and cost-effectiveness to maintain (Mizuno et al., 2010). α-Syn expression in *Drosophila* can cause dopaminergic neuron loss, Lewy body-like inclusion body formation and locomotor dysfunction (Feany and Bender, 2000) making this invertebrate an attractive model to study PD.

In order to extend these analyses further to another animal model of α-syn aggregation, we have used the nematode worm *Caenorhabditis elegans* (*C. elegans*), which is characterized by a simple anatomy, short lifespan, and well-established genetics. For these reasons, this system has become a powerful tool in biomedical research, in particular for genetic (Dillin et al., 2002; Jorgensen and Mango, 2002; Morley et al., 2002; Lee et al., 2003; Nollen et al., 2004; Hamilton et al., 2005; Kim and Sun, 2007; Sarin et al., 2008; Van Ham et al., 2008, 2010; Van der Goot et al., 2012) and drug (Wu et al., 2006; Alavez et al., 2012; Habchi et al., 2016; Perni et al., 2017a, 2018c; Limbocker et al., 2019). In particular, worms expressing the A30P and A53T variants in dopaminergic neurons have been reported in a previous study (Kuwahara et al., 2006) exhibiting accumulation of α-syn in the cell bodies and neurites of dopaminergic neurons, failure in modulation of locomotory rate in response to food, and reduction in neuronal dopamine content. These cell-specific dysfunctions caused by accumulation of α-syn appear relevant to the genetic and compound screenings aiming at the elucidation of pathological cascade and therapeutic strategies for PD. Further models were developed to evaluate the effect of the α-syn overexpression in other cell tissues, such as the muscle cells (Van Ham et al., 2008), and have been widely used for genetic screenings (Van der Goot et al., 2012).

Building on this evidence, we aimed to create a worm transgenic model expressing A30P and A53T variants that could be applied also in high-throughput drug screening studies. To achieve this goal, we chose to overexpress the A30P and A53T variants in the big muscle cells of the worms to affect directly the worms motility. We were then able to directly monitor the impact of the α-syn mutational variants on the worm fitness by using our recently developed high-throughput machine vision system (Perni et al., 2018a,b). We describe here the creation of two *C. elegans* models of familial PD that express the human α-syn gene carrying the A30P and the A53T mutations, indicated here as PD_A30P_ and PD_A53T_, respectively. We used for comparison a well-characterized PD worm model, which is based on the overexpression of wild type α-syn tagged with the yellow fluorescent protein (YFP) in the muscle cells of the worms (Van Ham et al., 2008), indicated here as PD_WT_. In order to facilitate a direct comparison between the variants and the wild-type worms, we also generated a fusion construct of YFP with the A30P and A53T variants. The control healthy worms, which express only YFP in the big muscle cells, are indicated here as the YFP strain. The PD_WT_ reference model, in which the presence of α-syn causes characteristic phenotypic changes (Van Ham et al., 2008), has been used successfully to probe the nature of a range of neurodegenerative conditions and has been employed in high-throughput screens to identify genes and to search for α-syn-related phenotypes (Van Ham et al., 2010; Van der Goot et al., 2012).

The aggregation of α-syn has been shown to be enhanced dramatically by its binding to lipid membranes (Flagmeier et al., 2016), and we recently showed that disrupting this interactions can be achieved with small molecules (Perni et al., 2017b; Limbocker et al., 2020). We reported in particular that the aminosterol squalamine (Moore et al., 1993; Rao et al., 2000; Zasloff et al., 2001, 2011), and related compounds (Perni et al., 2018c) can inhibit the binding of α-syn to membranes, reduce the initiation of its aggregation *in vitro*, and decrease its toxicity in human neuroblastoma cells and in a *C. elegans* model of PD (Perni et al., 2017b). Squalamine is currently in clinical trials for the treatment of symptoms associated with PD (ClinicalTrials.gov Identifier: NCT03781791). In order to explore the value of these worm models in the context of familial forms of PD, we used our recently developed high-throughput screening strategy (Perni et al., 2017b, 2018a,b) to investigate the effects of squalamine on the A30P and A53T worm variants developed in this study. We complemented these studies by also administering to our worm models an antibody that binds to a region of α-syn that has previously been identified to play a key structural role in its membrane-associated aggregation (Fusco et al., 2016) and to mitigate the toxicity of α-syn oligomers (Fusco et al., 2017).

## Results

### Effects of the Mutations on the Fitness of the PD_A30P_ and PD_A53T_ Worms

**Figure 1:**
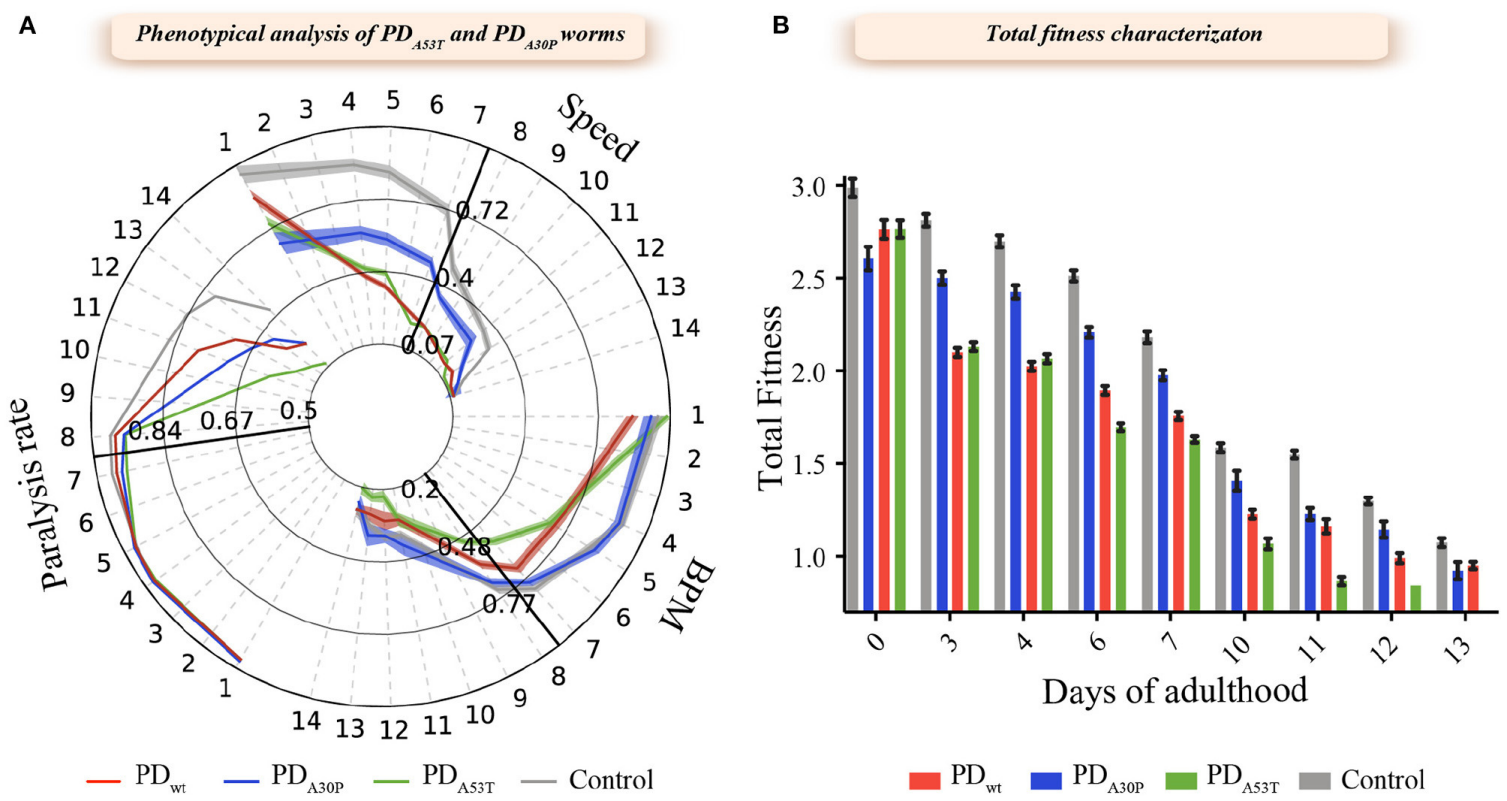
Behavioral characterization of the PD_A30P_ and PD_A53T_ worm strains. **(A)** Three common readouts of worm fitness were investigated for each strain using an automated worm tracking procedure (Perni et al., 2017b). The results are presented with a behavioral time-course map of PD_A30P_ and PD_A53T_ worms over 14 days of adulthood. The speed of movement, number of body bends per minute (BPMs), and the rate of paralysis were followed during aging; data are normalized with respect to day 1 to illustrate the progressive decline in all readouts. PD worms overexpressing wild-type α-syn:YFP in the body-wall muscle cells (PD_WT_) were used as a positive control, while worms expressing only YFP were used as negative healthy controls (Control). Shadowed areas represent standard errors of the mean (SEM). **(B)** The rate of body bends, speed and paralysis rate were combined into a single score of total fitness and evaluated during the duration of the experiment. For each experiment, about 1,000 worms were analyzed and each experiment was carried out in triplicate; one representative experiment of three experiments is shown. At each time point, the mutant worms exhibited lower fitness (*p* < 0.0005) when compared to healthy worms; error bars represent the standard error of the mean (SEM); the statistical significance was assessed using the 2-way ANOVA method with Dunnett's multiple comparison test.

We first characterized the behavior of the PD_A30P_ and PD_A53T_ worms in combination with the definition of the aggregation profile of α-syn in these two strains, and compared the results with the corresponding data for PD_WT_ worms. We observed that well-established behavioral characteristics, such as body bends per minute (BPMs) (Van Ham et al., 2008; Gidalevitz et al., 2009; Van der Goot et al., 2012; Habchi et al., 2016, 2017; Aprile et al., 2017; Perni et al., 2017a,b), speed of movement (Morley et al., 2002; Van Ham et al., 2008; Gidalevitz et al., 2009) and paralysis rate (Link, 1995; Lublin and Link, 2013; Perni et al., 2017b), were all affected to different extents by the overexpression of the A30P and A53T variants (Figure 1). In particular, the PD_A30P_ worms showed reduced speed of movement and an increased paralysis rate, but no relevant change in the frequency of body bends (BPMs) (Figure 1A). By contrast, both the frequency of body bends and speed of movement were found to be significantly decreased (*P* < 0.005) in the PD_A53T_ worms relative to the YFP and PD_WT_ worms. PD_A53T_ worms also showed a higher level of reduction in bend frequency and speed of movement, and higher paralysis rate, when compared with the PD_A30P_ and PD_WT_ worms (Figure 1A). These results suggest that the observed effects of the modified protein are related to different mechanisms of induced dysfunction compared to wild-type protein. Despite the observed phenotypical differences, the levels of expression of α-syn present in the PD_WT_, PD_A30P_ and PD_A53T_ worms were found to be similar (Supplementary Figure 1).

**Figure 2:**
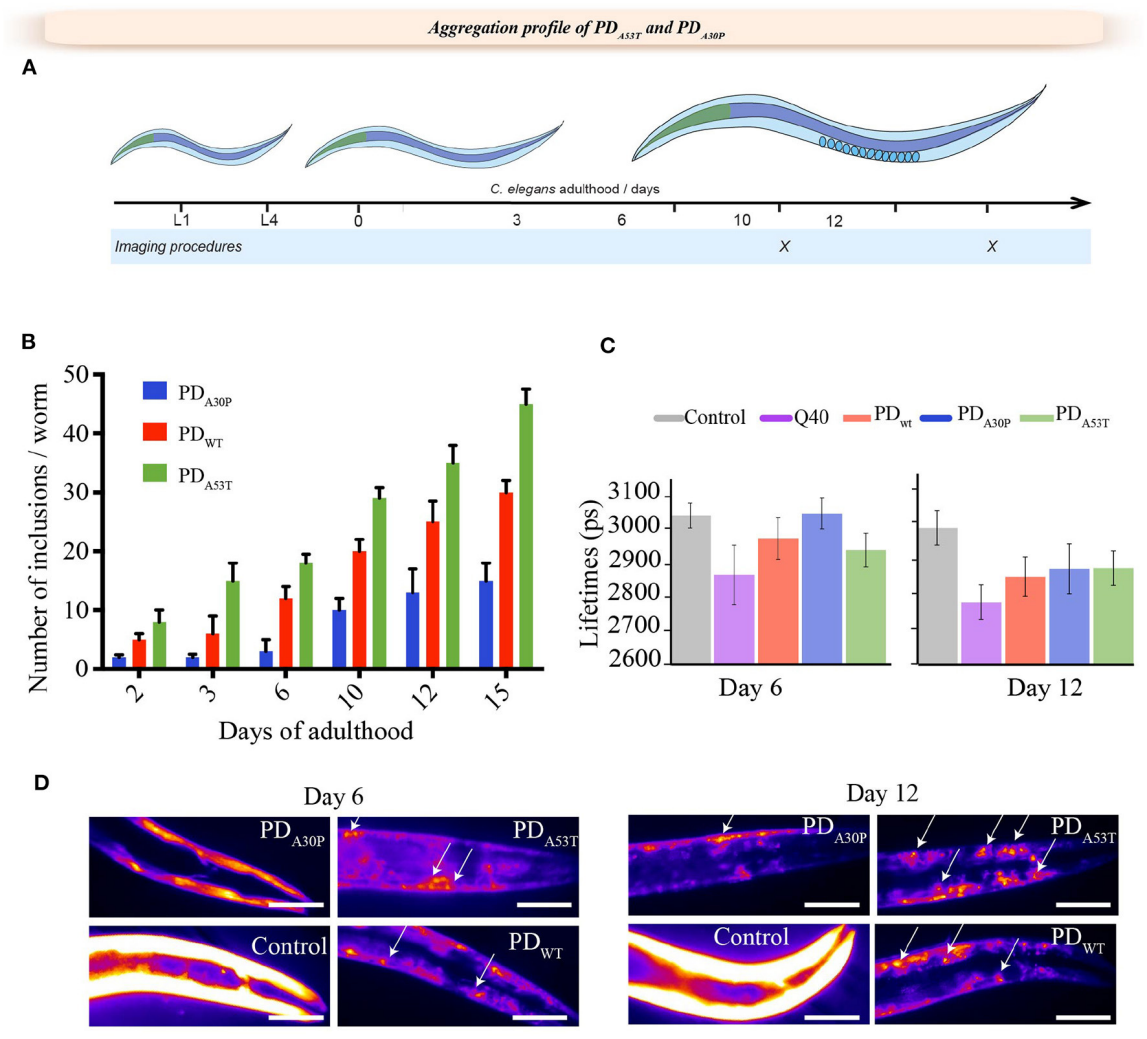
Age-dependent formation of inclusions in PD_A30P_ and PD_A53T_ worms. **(A)** Schematic description of the lifespan of *C. elegans*. Days 6 and 12 of adulthood are reported here as representative for the formation of α-syn inclusions *in vivo*. **(B)** Quantification of the number of α-syn inclusions in PD_A30P_ and PD_A53T_ worms, compared to PD_WT_ worms (Van Ham et al., 2008). **(C)** PD_A30P_ and PD_A53T_ amyloid-like aggregation profiles measured using TG-FLIM (Laine et al., 2019), (left) day 6 and (right) day 12. The average fluorescence lifetime (in ps) was measured for PD_A30P_ (blue) and PD_A53T_ (green) worms, and compared to control worms (gray) and PD_WT_ worms (red) (Van Ham et al., 2008), and to Q40 (pink) polyglutamine worms, which have a high propensity to form amyloid-like aggregates (Morley et al., 2002). The FLIM analysis shows no amyloid-like features in the α-syn inclusions in PD_A30P_ worms at day 6 of adulthood, unlike PD_A53T_, PD_WT_ and Q40 worms. A statistically significant increase in amyloid-like aggregation was observed in all the strains after day 12 of adulthood (insets) (*p* < 0.05). For each experiment, 25 worms were analyzed. **(D)** Representative images showing the inclusion profile in PD_A30P_ and PD_A53T_ worms and compared to PD_WT_ worms and YFP controls at day 6 and 12 (Van Ham et al., 2008).

To assess the influence of the amino acid substitutions on the behavior of the different worm strains, we first calculated the total fitness values, in both cases defined by a sum of the behavioral parameters, and compared these values to these of the PD_WT_ worms (Perni et al., 2017b, 2018b). The total fitness score is calculated as the sum of the frequency of body bend, speed of movement, and paralysis rate, normalized by the value at day 1. In the case of the PD_A30P_ worms, we observed a moderate reduction in the fitness value compared to the control YFP worms (Figure 1). A comparison of PD_A53T_ worms with control YFP worms after day 6 of adulthood, however, demonstrated a significantly increased level of dysfunction that correlates with the higher degree of formation of inclusions in the former model (Figures 2A,B). This dysfunction appeared also more extensive than the one observed in the case of PD_WT_ and YFP control worms. This observation is consistent with the reported effect of the A53T mutation, which is to increase the aggregation of α-syn *in vitro*. In particular, in these latter experiments we found that the lipid-induced nucleation and fibril amplification steps that result in the formation of an increased number of new aggregates, are accelerated for the A53T variant compared to the wild-type protein (Flagmeier et al., 2016), in accord with the *in vivo* findings.

### Effects of A30P and A53T Mutations on the *in vivo* Aggregation of α-syn

Protein aggregation can be studied *in vitro* by means of a range of well-established biophysical techniques (Arosio et al., 2014; Buell et al., 2014; Galvagnion et al., 2015; Flagmeier et al., 2016; Habchi et al., 2016). As direct observations of the nature and kinetics of the aggregation processes taking place *in vivo* provide opportunities to extend such findings to physiological conditions (Morley et al., 2002; Nollen et al., 2004; Van Ham et al., 2008; Van der Goot et al., 2012; Habchi et al., 2016), we investigated here the development of aggregates in both PD_A30P_ and PD_A53T_ worms, and compared their aggregation profiles with those of PD_WT_ worms (Van Ham et al., 2008) (Figure 2). We observed that until day 6 of adulthood, inclusions in PD_WT_ and PD_A30P_ worms showed a diffused fluorescence intensity pattern similar to that of the control worms expressing only YFP, indicating that they are largely unstructured and diffuse (Figures 2B,D). After that, we could observe the presence of more well-defined aggregates (Figures 2B,D).

We further analyzed the nature of the aggregates using fluorescence lifetime imaging (FLIM), a technique that enables the specific kinetics of protein aggregation to be followed *in vivo* (Schierle et al., 2011; Laine et al., 2019). This methodology is based on a fluorophore covalently linked to the amyloidogenic protein of interest (Schierle et al., 2011). We have previously shown that a reduction in the fluorescence lifetime of a reporter fluorophore, such as YFP, correlates with the degree of aggregation of the protein to which it is attached, and that this effect provides a quantitative measure of the degree of protein aggregation *in vitro*, in live cells and in *C. elegans* (Schierle et al., 2011). This decrease in lifetime is thought to be associated with the fluorescence energy transfer to electronic states associated with the amyloid structure (Schierle et al., 2011). Taken together, these results indicate that the process of aggregation *in vitro* and the ability of A53T to induce dysfunction in nematode worms from day 6 of adulthood is significantly faster than that of A30P and that of the wild-type protein, as also observed *in vitro* (Flagmeier et al., 2016).

### Effects of the Aminosterol Squalamine on PD_A30P_ and PD_A53T_ Worms

The aminosterol squalamine (Rao et al., 2000; Zasloff et al., 2001, 2011) was shown to be an effective inhibitor of *in vitro* (Perni et al., 2017b), and to suppress α-syn-mediated toxicity in neuronal cells and in a *C. elegans* model of PD (Perni et al., 2017b). The primary mode of action of this compound is the displacement of monomeric and oligomeric forms of α-syn from lipid membranes both *in* lipid vesicles and in cell membranes.

**Figure 3:**
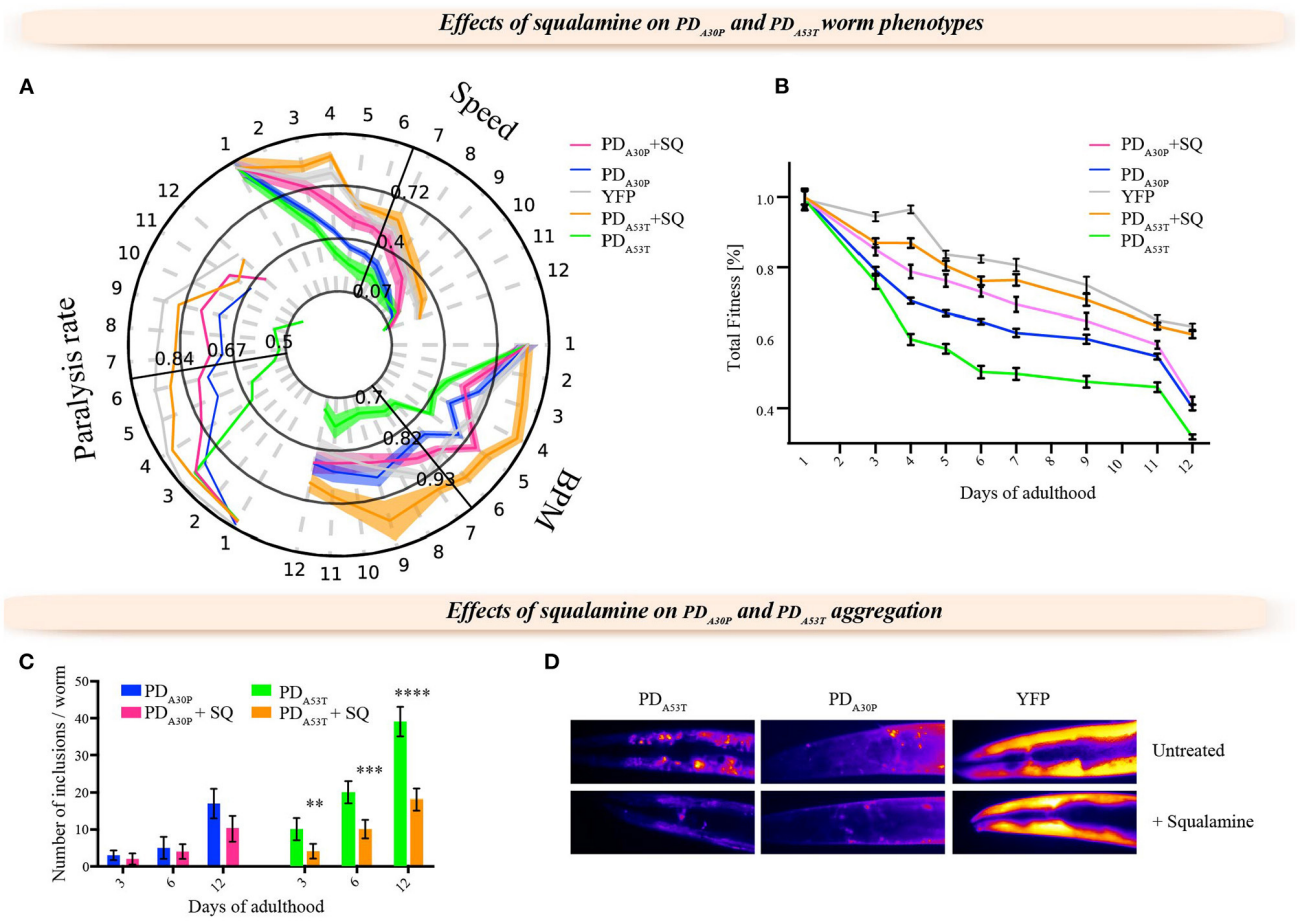
Effects of squalamine on the formation of α-syn inclusions and the fitness of PD_A30P_ and PD_A53T_ worms. **(A)** Behavioral time-course map reporting thee rate of body bends, swimming speed and paralysis rate for PD_A30P_ and PD_A53T_ worms exposed to 10 μM squalamine. **(B)** Calculation of the total fitness corresponding to the three readouts in **(A)** (Perni et al., 2017b, 2018b). The effects of squalamine on control worms were negligible (Perni et al., 2017b), and only a small protective effect could be observed in PD_A30P_, while strong protective effects were observed in PD_A53T_ worms consistently with the effects on the PD_WT_ worms (Perni et al., 2017b). Each experiment was carried out in triplicate, and about 1,000 worms were analyzed in each replicate; one representative experiment of the three is shown. **(C)** Quantification of the number of inclusions in PD_A30P_ and PD_A53T_ worms with or without squalamine; squalamine reduced significantly the number of inclusions in PD_A53T_ worms ^**^P ≤ 0.01, ^***^P ≤ 0.001 and ^****^P ≤ 0.0001. **(D)** Representative images showing a substantial decrease in the number of α-syn inclusions in the presence of squalamine in PD_A53T_ worms. In order to show the inclusions more clearly, the image focus on only the heads of the worms, although the quantification was carried out using the whole worms. This result is consistent with previous observations for PD_WT_ worms (Perni et al., 2017b). In the YFP worms, the expression pattern is not significantly affected while in PD_A30P_ worms the number of inclusions appears mildly reduced. All measurements were carried out at day 12 of adulthood.

In order to investigate the use of the PD_A30P_ and PD_A53T_ worm models and obtain insights into the nature of familial forms of PD, we administered squalamine to both PD_A30P_ and PD_A53T_ worms by evaluating its effect on the rate and degree of aggregation of the α-syn variants within the worms. We observed that squalamine had a smaller effect on the behavior of the PD_A30P_ compared to PD_WT_ worms, but increased substantially the rate of body bends, speed of movement and the paralysis rate of the PD_A53T_ worms, as found with PD_WT_ worms, and effectively restored their behavior to that of the control YFP worms (Figure 3A). These results are illustrated further by comparison of the values of the total fitness in each case (Figure 3B).

We next investigated the effects of squalamine on the formation of aggregates of α-syn in the PD_A53T_ and PD_A30P_ worms (Figures 3C,D). In the presence of squalamine, the number of α-syn inclusions was reduced in the PD_A53T_ worms, but less so in the PD_A30P_ worms, despite the fact that the levels of α-syn expression in PD_A30P_ and PD_A53T_ worms in the presence of squalamine were similar to that of the PD_WT_ animals (Supplementary Figure 1). We also found that squalamine did not significantly affect the lipid-induced aggregation process of the A30P variant *in vitro* (Supplementary Figure 2), while it did so for the wild-type protein (Perni et al., 2017b). As the A30P variant has been shown to have reduced binding to cell membranes (Jo et al., 2002), the observation of the reduced effects of squalamine in the A30P variant compared to the A53T and wild-type variants further supports the conclusion that the mechanism of action of this small molecule *in vivo* is mediated by its competitive binding to cell membranes (Perni et al., 2017b).

### Effects of an Antibody Targeting the N-Terminal Region of α-syn on PD_A30P_ and PD_A53T_ Worms

**Figure 4:**
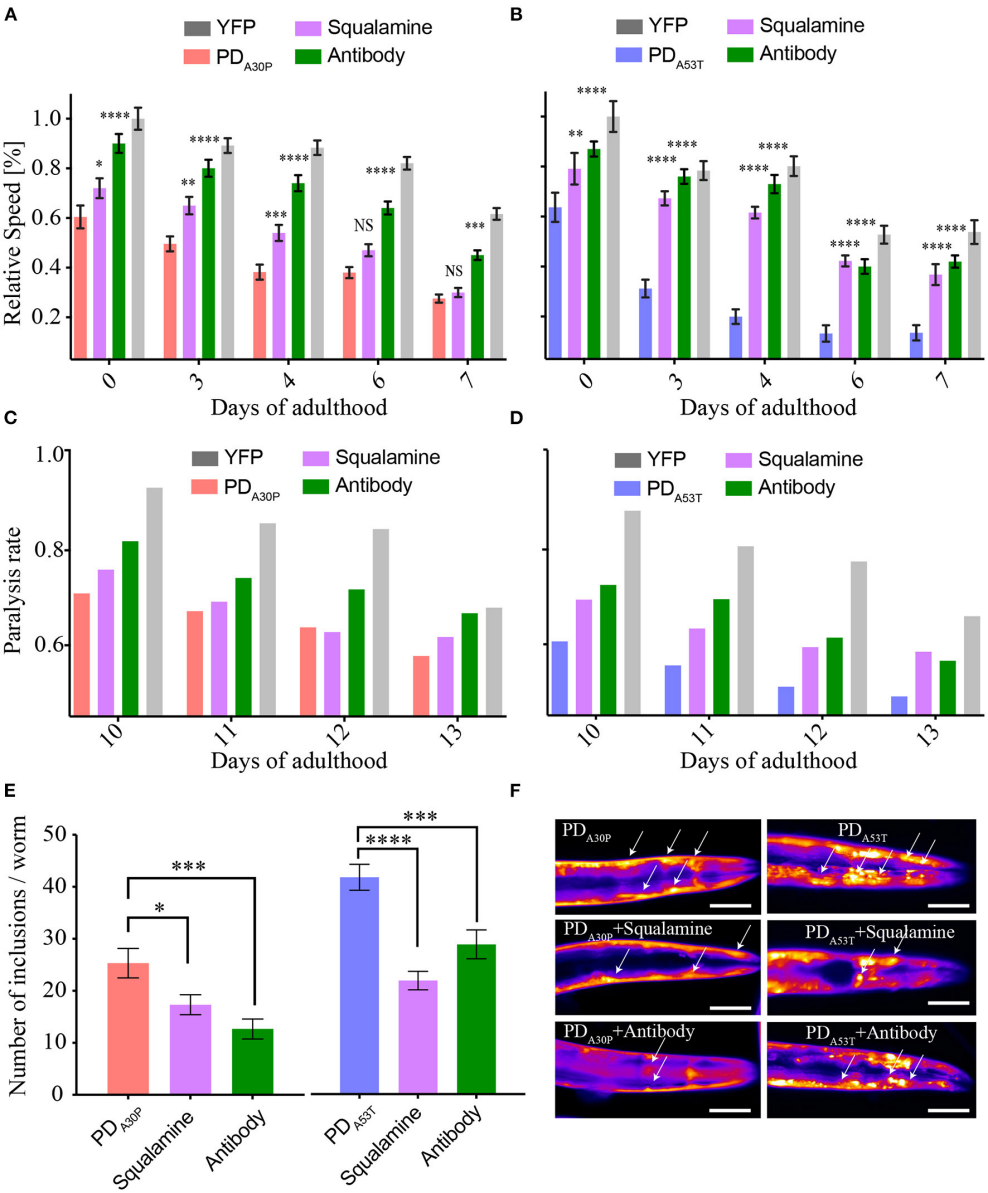
Comparison of the effects of the antibody and squalamine administration to PD_A30P_ and PD_A53T_ worms. **(A,B)** At a concentration of 10 μM, the antibody rescues the motility dysfunction (relative speed) induced by the over-expression of A30P, with squalamine having a slightly smaller effect. The worm motility is also rescued in the A53T worms by the antibody and squalamine to similar extents. Errors represent the standard error on the mean (SEM) ^*^P ≤ 0.05, ^**^P ≤ 0.01, ^***^P ≤ 0.001 and ^****^P ≤ 0.0001. **(C,D)** Paralysis rate, reported as the fraction of worms that are mobile, corresponding to the time points shown in **(A,B)**; the variability between biological replicates is in the 2–9% range, and the variability between technical replicates is in the 1–4% range. **(E,F)** Number of inclusions at day 12 of adulthood ^*^P ≤ 0.05, ^***^P ≤ 0.001 and ^****^P ≤ 0.0001. The scale bar indicates 80 μm.

In order to probe further the behavior of the various α-syn forms in *C. elegans*, we administered to the PD_A30P_ worms a previously described antibody (Fusco et al., 2016) that binds to the N-terminal region of the α-syn sequence (residues 1–25). This region was found to play a key structural role in the membrane-associated aggregation of α-syn (Fusco et al., 2016) and in the toxicity of α-syn oligomers (Fusco et al., 2017). When the antibody was incubated with PD_A30P_, a reduction in the toxicity that resulted from the overexpression of α-syn was observed, to an extent similar to that observed in the case of PD_WT_ (Perni et al., 2017b). The effect of the antibody on PD_A30P_ appeared to be slightly greater than that induced by squalamine (Figure 4), which could be a result of the more specific action of the antibody in suppressing the toxicity associated with overexpression of α-syn molecules in the worms, particularly showing a direct interaction with the exposed N-terminal region of α-syn in the oligomeric species. By contrast, the antibody was observed to exert effects similar to those resulting from the addition of squalamine on the toxicity observed in PD_A53T_ worms, as shown by an increase in the rate of body bends and in the speed of movement, and by a decrease in the paralysis rates (Figure 4).

## Discussion and Conclusions

We have created and characterized two *C. elegans* strains, PD_A30P_ and PD_A53T_, expressing the A30P and A53T mutational variants, respectively, which are associated with familial forms of PD. We have then demonstrated that these two mutational variants affect the worms in different ways and to different extents. The expression of the A30P species was shown to reduce specifically certain aspects of worm behavior, notably speed of swimming, compared with the wild-type protein. Overexpression of the A53T mutation, however, had a more dramatic effect than that found for the wild-type protein, and the worms expressing this variant behaved in a dysfunctional manner at a significantly younger age than did those expressing the A30P or the wild-type forms. Overall, the expression of the A53T variant resulted in a more significant decrease in the bends, and speed of movement compared with the A30P and wild-type proteins. We note that worms expressing the A30P and A53T variants in dopaminergic neurons exhibited a less severe phenotype (Kuwahara et al., 2006), suggesting that the overexpression of α-syn in muscle cells may lead to increased toxicity through additional mechanisms with respect to those involved in PD.

These findings are broadly consistent with the measurement and analysis of the kinetics of aggregation *in vitro*. In particular, the observation that the expression of the A30P variant alters the phenotype of the worms moderately compared to the dysfunction associated with the expression of the wild-type protein, is in agreement with the findings that the overall rate of aggregation is only mildly affected for the A30P variants when compared to wild-type *in vitro* (Conway et al., 2000; Flagmeier et al., 2016). Initiation of the *in vitro* aggregation process, however, has been found to be faster for the A53T variant than for A30P or wild-type protein (Flagmeier et al., 2016), an observation consistent with more rapid decline of the fitness of the PD_A53T_ related to the PD_A30P_ or PD_WT_ worms. Taken together, these results are particularly interesting in the context of the clinical manifestations of the A30P and A53T mutations, where patients with the A30P mutation appear generally to exhibit similar age of onset and rate of disease progression to those suffering of sporadic PD, while patients carrying the A53T mutation generally exhibit an earlier age of onset and have a more rapid rate of progression of the disease (Polymeropoulos et al., 1997; Krüger et al., 2001; Schiesling et al., 2008).

The degree of dysfunction of the *C. elegans* model expressing human α-syn has recently been shown to be reduced substantially by the administration of squalamine (Perni et al., 2017b), a naturally active aminosterol, and we have shown here that this small molecule decreases the amount of fitness reduction and aggregation to a lower extent in the PD_A30P_ than in the PD_WT_ worms, but has a more substantial effect in the PD_A53T_ worms, which is similar to that observed in PD_WT_ worms. These results are consistent with the finding that squalamine reduces the membrane-associated initiation of the aggregation of α-syn by displacing it from the surfaces of lipid bilayers (Perni et al., 2017b). In addition, we observed that an antibody targeting the N-terminal region of the protein, which plays a key role in both the aggregation process and the induction of cellular toxicity by α-syn oligomers, was also protective in PD_WT_ and PD_A53T_ worms, while less so in PD_A30P_ worms. Overall, this analysis provides support to the strategy of reducing the binding of α-syn to lipid membranes as a potential therapeutics strategy for PD.

## Materials and Methods

Extended experimental procedures are described in **SI Materials and Methods**. *In vitro* kinetic experiments and purifications of wild type and mutant α-syn were carried out as previously indicated (Flagmeier et al., 2016). TG-FLIM imaging was carried out on a home-built microscopy platform described elsewhere (Schierle et al., 2011; Laine et al., 2019). *In vivo* experiments were carried out by using a well-studied *C. elegans* model of PD (Link, 1995) and custom made A53T and A30P strains. Microinjection was used to create new transgenic strains and standard conditions were used for the propagation of *C. elegans* (Brenner, 1974). Squalamine was synthesized as previously described (Zhang et al., 1998) and automated behavioral assays were carried out as previously described (Perni et al., 2017b, 2018a,b). Measurements on inclusions *in vivo* were performed using ImageJ software as previously described (Van der Goot et al., 2012; Perni et al., 2017b). Western blot analysis was carried out as previously described (Limbocker et al., 2019). The transduction of the antibody was carried out as previously reported (Aprile et al., 2017; Perni et al., 2017a).

## Data Availability Statement

The original contributions presented in the study are included in the article/Supplementary Material, further inquiries can be directed to the corresponding author/s.

## Ethics Statement

Ethical review and approval was not required for the animal study because *C. elegans* does not require ethical review and approval.

## Author Contributions

AG, TH, KT created the *C. elegans* strains. MP, RLi, and MM characterized the *C. elegans* strains. MP and RLi carried out the measurements of the measurements of the effects of squalamine. PF carried out the *in vitro* experiments. RLa and MP carried out the FLIM experiments. MP carried out the antibodies testing. RLi and FA carried out the western blotting and subsequent analysis. GF, SCh, and AD were involved in the antibody design. MP, FA, PF, RLi, MM, EN, MV, and CD designed the study. All the authors were involved and contributed in the writing and revision of the manuscript.

## Conflict of Interest

MZ and DB are inventors in a patent for the use of squalamine in the treatment of PD. CD, MV, SCo, and TK are co-founders, and MP is an employee of Wren Therapeutics, which is independently pursuing inhibitors of protein misfolding and aggregation. The remaining authors declare that the research was conducted in the absence of any commercial or financial relationships that could be construed as a potential conflict of interest.
