# Supplementary material for: Comparative Studies in the A30P and A53T α-Synuclein C. elegans Strains to Investigate the Molecular Origins of Parkinson's Disease
Source: Front Cell Dev Biol. 2021 Mar 22;9:552549. doi: 10.3389/fcell.2021.552549 (PMC8019828; doi:10.3389/fcell.2021.552549)
Supplement: Supplementary file 1 [file Table_1.DOCX]

**Supporting Information**

**Materials and Methods**

**Growth media**. Standard conditions were used for the propagation of *C. elegans* (Brenner, 1974). Briefly, the animals were synchronized by hypochlorite bleaching, hatched overnight in M9 (3 g/l KH_2_PO_4_, 6 g/l Na_2_HPO_4_, 5 g/l NaCl, 1 µM MgSO_4_) buffer, and subsequently cultured at 20 °C on nematode growth medium (NGM) (CaCl_2_ 1mM, MgSO_4_ 1mM, cholesterol 5 μg/ml, 250 µM KH_2_PO_4_ pH 6, Agar 17 g/L, NaCl 3 g/l, casein 7.5 g/l) plates seeded with the *E. coli* strain OP50. Saturated cultures of OP50 were grown by inoculating 50 mL of LB medium (tryptone 10 g/l, NaCl 10 g/l, yeast extract 5 g/l) with OP50 and incubating the cultures for 16 h at 37 °C. NGM plates were seeded with bacteria by adding 350 µl of saturated OP50 to each plate and leaving the plates at 20 °C for 2-3 days. On day 3 after synchronization, the animals were placed on NGM plates containing 5-fluoro-2'deoxy-uridine (FUDR) (75 µM, unless stated otherwise) to inhibit the growth of offspring.

***C. elegans* strains**. All the worm strains were custom made or obtained from the *C. elegans* Genetic Centre (CGC) (MI, USA). The following strains were used: zgIs15 [P(unc-54)::α-syn::YFP]IV (OW40). In OW40, α-syn fused to YFP forms inclusions that increase in number and size during the ageing of the animals, up to late adulthood (Day 17) (Link, 1995); rmIs126 [P(unc-54)Q0::YFP]V (OW450). In OW450, YFP alone is expressed and remains diffusely localized throughout ageing (Cohen et al., 2013; Scherzinger et al., 1999). rmIs133 [unc-54p::Q40::YFP]. AM141 animals show a soluble Q40::YFP distribution in body wall muscle cells immediately after hatching. As these worms age the rapid formation of aggregate foci is observed. When they reach adulthood, AM141 animals show an entirely Q40::YFP aggregated phenotype(Morley et al., 2002). OW43 zgIs17[P(unc-54)::αsynA30P::YFP] II; OW433 zgIs52[P(unc54)::αasyn(A53T)::YFP] and OW450 rmIs126[P(unc-54)::Q0::YFP] V.

**Constructs**. To create P(unc-54)::asynA30P::YFP (pENG2) and P(unc54):: :α syn(A53T)::YFP (pENG4), site-directed mutagenesis was used with [P(unc-54):: :αsyn::YFP (pENG1) as a template.

**Creation of transgenic strains.** Microinjection was used to create new transgenic strains. 1 ng/μl plasmid DNA was injected into N2 animals, and integrated by γ irradiation, and the animals were outcrossed at least 4 times with untreated N2 animals. The YFP tag was chosen as a fusion construct and fused to the α-syn as a selection marker.

**Treatment with squalamine**. Squalamine was synthesized as previously described (Zhang et al., 1998). NGM plates containing fluorodeoxyuridine FUDR (75 µM, unless stated otherwise), were seeded with 350 µL OP50 culture and incubated for up to 3 days at room temperature. Afterwards these plates were seeded with aliquots of squalamine dissolved in water, at a final concentration of 10 μM. The plates were then placed in a laminar flow hood at room temperature to dry and the worms were transferred to plates coated with squalamine at larval stage L4.

**Automated motility assay on agar plates**. Automated behavioural assays were carried out as previously described (Perni et al., 2017b; Perni et al., 2018; Perni et al., 2018)^.^ All *C. elegans* populations were cultured at 20 °C and developmentally synchronized from a 4 h egg-lay. At 64-72 h post egg-lay (time zero) individuals were transferred to FUDR plates, and body movements were assessed over the times indicated. At different ages, the animals were washed off the plates with M9 buffer and spread over an OP-50 unseeded 6 cm plate, after which their movements were recorded at 30 fps using a recently developed microscopic procedure for 30 s or 1 min (Perni et al., 2017b). Up to 200 animals were counted in each experiment unless otherwise stated. The videos were analyzed using a custom made tracking code (Perni et al., 2017b; Perni et al., 2018; Perni et al., 2018).

**Quantification of inclusions**. To monitor the number of inclusions, individual animals were mounted on 2% agarose pads, containing 40 mM NaN_3_ as an anesthetic, on glass microscope slides for imaging. For quantification of the number of inclusions in α-syn:YFP and mutated variant strains, only the frontal region of the worms was considered (Takamori et al., 2006). The numbers of inclusions in each animal were quantified using a Leica MZ16 FA fluorescence dissection stereomicroscope (Leica Microsystems, Wetzlar, Germany) at a nominal magnification of 20X or 40X, and images were acquired using an Evolve 512 Delta EMCCD Camera, with high quantum efficiency (Photometrics, Tucson, AZ, USA). Measurements on inclusions were performed using ImageJ software as previously described (Perni et al., 2017b; Van der Goot et al., 2012). At least 50 animals were examined under each condition, unless stated otherwise. All experiments were carried out in triplicate. The Student's t-test was used to calculate p values, and all tests were two-tailed unpaired unless otherwise stated.

**Western blot analysis.** For comparison of α-syn levels in the different strains, ca. 3,000 adult worms were collected in S-basal (Brenner, 1974) in triplicate and frozen in liquid N_2_. Samples were then extracted in urea/SDS buffer (8 M urea, 2% SDS, 50 mM Tris, 1× proteinase inhibitors) (Roche, Basel, Switzerland) and disrupted via sonication. Samples at the appropriate concentration were added to NuPAGE LDS Sample Buffer (1×) and NuPAGE Sample Reducing Agent (1×) (Life Technologies, Carlsbad, CA, USA) and heated at 70 °C for 10 min. The resulting material was resolved via NuPAGE Novex 4–12% Bis-Tris Protein Gels (Life Technologies) and then transferred to nitrocellulose membranes, using an iBlot Dry Blotting System (Life Technologies, Carlsbad, CA, USA). Prior to transferring, the gels were incubated at room temperature in 10% ethanol for 10 min, and the membrane was then blocked for 1 h at room temperature in blocking solution (PBS, 0.1% tween, 5% milk), washed (PBS, 0.1% tween), and incubated overnight at 4 ˚C with either the SYN-1 anti-α-syn antibody, clone 42 (BD Biosciences, Franklin Lakes, NJ, USA) at a dilution of 1:500, or the anti-α-tubulin antibody, clone B-5-1-2 (Sigma-Aldrich, St. Louis, MO, USA) at a dilution of 1:1000. After washing, an Alexa 488-conjugated secondary antibody, A11029 (Life Technologies, Carlsbad, CA, USA), was incubated for 1 h at room temperature. Membranes were then washed and measured for fluorescence using a Typhoon Trio Imager (GE Healthcare, Chicago, IL, USA).

**Time-gated fluorescence lifetime imaging microscopy (TG-FLIM) imaging**: TG-FLIM imaging was carried out on a home-built microscopy platform that permits fast measurements of lifetime to be recorded for every image pixel as described elsewhere (Laine et al., 2018; Schierle et al., 2011) Briefly, the TG-FLIM system was set up on an Olympus IX83 frame with a a super-continuum laser source Fianium SC400-4(Kaminski et al., 2008). Each fluorescence image was time-gated using a high-rate imager (HRI, Kentech, UK) and a camera sensor (PCO pixelfly, USB, PCO, Germany). Imaging of worm heads was performed using a 10X objective (Olympus PlanFLN 10X NA 0.3). The excitation wavelength was selected as 516 nm (10 nm bandwidth) and the fluorescence was detected using a 550/49 (Semroc) filter. The instrument response function (IRF) was measured by acquisition of 1 mM solutions of erythrosin B (Sigma-Aldrich) solution in water. FLIM reconstruction was performed using the FLIMfit (Warren et al., 2013) package from the Open Microscopy Environment (OME) (Linkert et al., 2010). Data analysis was performed by removing the background signal from the images and using spatially-varying IRF reference reconvolution with a lifetime of ~90 ps (Schierle et al., 2011). The data were fitted to a single exponential decay on a single-pixel basis (Schierle et al., 2011).

**Transduction protocol.** Transduction was carried out as previously reported (Aprile et al., 2017; Perni et al., 2017a). Briefly, about 500 PD_A30P_ worms were incubated in M9 with 128001 and 128003 (Synaptic Systems, Goettingen, Germany) and 40 μl PulsIn (40-70 μM) (PolyPlus Tranfection SA, Illkirch-Graffenstaden, France) in a final volume of 1 ml for 6 h. Motility screening was carried out 24 h after transduction. All experiments were carried out in triplicate. As a control we used worms treated with empty vesicles to take into consideration possible effects of the lipids on the behavior of the worms.

**Quantitative real time PCR (qPCR).** To assess the levels of α-syn expression in the different *C. elegans* strains, the total RNA was isolated from the worms using TRIzol (#15596018, Invitrogen) according manufacturers protocol. Using a Nanodrop 2000 Spectrophotometer, the RNA concentration and quality were measured. From 1 µg total RNA, cDNA was made using the RevertAid H Minus First Strand cDNA Synthesis kit (#K1632, Life Technologies) using random hexamer primers. The Quantitative real-time PCR was performed using 2 µl of 10 times diluted cDNA using a Roche LightCycler 480 Instrument II (Roche Diagnostics). To detect cDNA amplification, the SYBR green dye (#172-5125, Bio-Rad) was used. The following protocol was used: 95 °C for 10 minutes, followed by 40 cycles at 95 °C for 15 seconds, 60 °C for 30 seconds and 72 °C for 15 seconds, the program ended with 95 °C for 5 seconds, 65 °C for 1 minute and 97 °C. Relative transcript levels were quantitated using a standard curve of pooled cDNA samples. Expression levels were normalized against *pmp-3,* an endogenous reference gene.

The primers used are

| Primer | Sequence |
| --- | --- |
| PMP-3.F3 | CACTCATCTCTATGACGACGTTTC |
| PMP-3.R3 | CACCGTCGAGAAGCTGTAGA |
| asyn_fw | TGGCTGCTGCTGAGAAAACC |
| asyn_rev | GCTCTTTGGTCTTCTCAGCC |
| YFP_fw | TGGACGGCGACGTAAAC |
| YFP_rev | TGGTGCAGATGAACTTCAGG |

**Measurement of aggregation kinetics**. Wild-type α-syn or its variants were incubated at the concentrations indicated and in the presence of 50 μM ThT and DMPS vesicles at 30 ◦C, respectively (Flagmeier et al., 2016b). The change in the ThT fluorescence signal was monitored using a Fluostar Optima or Polarstar Omega fluorescence plate reader (BMG Labtech, Aylesbury, UK) in bottom reading mode under quiescent conditions. Corning 96 well plates with half-area (3881, polystyrene, black with clear bottom) non-binding surfaces sealed with metal sealing tape were used for each experiment. For experiments involving shaking (orbital shaking for 300 s at 1,100 rpm), glass beads were introduced in the wells of the plate prior to the measurement and the change in the ThT fluorescence signal was monitored using a Polarstar Omega fluorescence plate reader. At the end of each aggregation experiment the concentrations of monomeric and fibrillar states of the protein were determined as described previously (Flagmeier et al., 2016b).

**Figure S1. Analysis of the α**-**syn expression levels in the different worm strains studied in this work.** Western blot analysis of the α-syn levels in the different strains (A,C), including upon administration of squalamine (D), and qPCR analysis of the α-syn levels in the different strains (B). Experiments were carried out in triplicate; for the quantification the averaged values of 3 independent replicas were used.

**Figure S2. Effect of squalamine on the aggregation of A30P *in vitro*.** The *in vitro* aggregation assay was carried out as previously described (Flagmeier et al., 2016a). Change in ThT fluorescence intensity when 100 µM monomeric wild-type or A30P α-syn was incubated in the presence of 100 µM DMPS vesicles and 50 µM ThT in 20 mM phosphate buffer (pH 6.5) under quiescent conditions at 30 °C. Squalamine was added to the solutions at increasing concentrations (black: 0 µM, dark blue: 1 µM, green: 2.5 µM, light blue: 5 µM, purple: 10 µM, red); two independent traces are shown for each concentration.

**Supplementary References**

Aprile, F. A., Sormanni, P., Perni, M., Arosio, P., Linse, S., Knowles, T. P. J., et al. (2017). Selective targeting of primary and secondary nucleation pathways in Aβ42 aggregation using a rational antibody scanning method. *Science Advances* 3, e1700488. doi:10.1126/sciadv.1700488.

AU Perni, M., AU Casford, S., AU Aprile, F. A., AU Nollen, E. A., AU Knowles, T. P. J., AU Vendruscolo, M., et al. (2018). Automated Behavioral Analysis of Large C. elegans Populations Using a Wide Field-of-view Tracking Platform. *J Vis Exp*, e58643.

Brenner, S. (1974). The genetics of Caenorhabditis elegans. *Genetics* 77, 71–94.

Cohen, S. I. A., Linse, S., Luheshi, L. M., Hellstrand, E., White, D. A., Rajah, L., et al. (2013). Proliferation of amyloid-β42 aggregates occurs through a secondary nucleation mechanism. *Proc Natl Acad Sci U S A* 110, 9758–9763. doi:10.1073/pnas.1218402110.

Flagmeier, P., Meisl, G., Vendruscolo, M., Knowles, T. P. J., Dobson, C. M., Buell, A. K., et al. (2016a). Mutations associated with familial Parkinson's disease alter the initiation and amplification steps of α-synuclein aggregation. *Proc Natl Acad Sci U S A* 113, 10328–10333. doi:10.1073/pnas.1604645113.

Flagmeier, P., Meisl, G., Vendruscolo, M., Knowles, T. P. J., Dobson, C. M., Buell, A. K., et al. (2016b). Mutations associated with familial Parkinson's disease alter the initiation and amplification steps of α-synuclein aggregation. *Proc. Natl. Acad. Sci. U.S.A.* 113, 10328–10333.

Kaminski, C. F., Watt, R. S., Elder, A. D., Frank, J. H., and Hult, J. (2008). Supercontinuum radiation for applications in chemical sensing and microscopy. *Applied Physics B* 92, 367.

Laine, R. F., Sinnige, T., Ma, K. Y., Haack, A. J., Poudel, C., Gaida, P., et al. (2018). Fast fluorescence lifetime imaging reveals the maturation process of α-synuclein aggregates in ageing &lt;em&gt;Caenorhabditis elegans&lt;/em&gt. *bioRxiv*, 414714.

Link, C. D. (1995). Expression of human beta-amyloid peptide in transgenic Caenorhabditis elegans. *Proc Natl Acad Sci U S A* 92, 9368–9372.

Linkert, M., Rueden, C. T., Allan, C., Burel, J.-M., Moore, W., Patterson, A., et al. (2010). Metadata matters: Access to image data in the real world. *J Cell Biol* 189, 777–782. doi:10.1083/jcb.201004104.

Morley, J. F., Brignull, H. R., Weyers, J. J., and Morimoto, R. I. (2002). The threshold for polyglutamine-expansion protein aggregation and cellular toxicity is dynamic and influenced by aging in Caenorhabditis elegans. *Proc Natl Acad Sci U S A* 99, 10417–10422. doi:10.1073/pnas.152161099.

Perni, M., Aprile, F. A., Casford, S., Mannini, B., Sormanni, P., Dobson, C. M., et al. (2017a). Delivery of Native Proteins into C. elegans Using a Transduction Protocol Based on Lipid Vesicles. *Scientific Reports* 7, 7380. doi:10.1038/s41598-017-13755-9.

Perni, M., Challa, P. K., Kirkegaard, J. B., Limbocker, R., Koopman, M., Hardenberg, M. C., et al. (2018). Massively parallel C. elegans tracking provides multi-dimensional fingerprints for phenotypic discovery. *J. Neurosci. Methods* 306, 57–67. doi:10.1016/j.jneumeth.2018.02.005.

Perni, M., Galvagnion, C., Maltsev, A., Meisl, G., Müller, M. B. D., Challa, P. K., et al. (2017b). A natural product inhibits the initiation of α-synuclein aggregation & suppresses its toxicity. *Proc. Natl. Acad. Sci. U.S.A.* 114, E1009–E1017.

Scherzinger, E., Sittler, A., Schweiger, K., Heiser, V., Lurz, R., Hasenbank, R., et al. (1999). Self-assembly of polyglutamine-containing huntingtin fragments into amyloid-like fibrils: Implications for Huntington’s disease pathology. *Proc Natl Acad Sci U S A* 96, 4604–4609. doi:10.1073/pnas.96.8.4604.

Schierle, G. S. K., Bertoncini, C. W., Chan, F. T. S., Van der Goot, A. T., Schwedler, S., Skepper, J., et al. (2011). A FRET sensor for non-invasive imaging of amyloid formation in vivo. *Chemphyschem* 12, 673–680. doi:10.1002/cphc.201000996.

Takamori, S., Holt, M., Stenius, K., Lemke, E. A., Grønborg, M., Riedel, D., et al. (2006). Molecular anatomy of a trafficking organelle. *Cell* 127, 831–846. doi:10.1016/j.cell.2006.10.030.

Van der Goot, A. T., Zhu, W., Vazquez-Manrique, R. P., Seinstra, R. I., Dettmer, K., Michels, H., et al. (2012). Delaying aging and the aging-associated decline in protein homeostasis by inhibition of tryptophan degradation. *Proc. Natl. Acad. Sci. U.S.A.* 109, 14912–14917.

Warren, S. C., Margineanu, A., Alibhai, D., Kelly, D. J., Talbot, C., Alexandrov, Y., et al. (2013). Rapid Global Fitting of Large Fluorescence Lifetime Imaging Microscopy Datasets. *PLoS ONE* 8, e70687. doi:10.1371/journal.pone.0070687.

Zhang, X., Rao, M. N., Jones, S. R., Shao, B., Feibush, P., McGuigan, M., et al. (1998). Synthesis of squalamine utilizing a readily accessible spermidine equivalent. *Journal of Organic Chemistry* 63, 8599–8603.
